# Editorial: Interplay between chronic pain and affective-cognitive alterations: shared neural mechanisms, circuits, and treatment

**DOI:** 10.3389/fphar.2025.1605670

**Published:** 2025-04-25

**Authors:** Luca Posa, Danilo de Gregorio, Serena Boccella, Kelly Smart

**Affiliations:** ^1^ Department of Biochemistry, Weill Cornell Medicine, New York, NY, United States; ^2^ IRCCS San Raffaele Scientific Institute, Milan, Italy; ^3^ Vita-Salute San Raffaele University, Milan, Italy; ^4^ Department of Experimental Medicine, Division of Pharmacology, University of Campania, Naples, Italy; ^5^ Brain Health Imaging Centre, Centre for Addiction and Mental Health, Toronto, ON, Canada

**Keywords:** chronic pain, cognition, emotional pain, neuronal circuits, therapeutics

Chronic pain, defined clinically as persisting for at least 3 months, is not merely a sensory phenomenon but a subjective experience shaped by affective and cognitive processing. Patients with persistent pain often suffer from emotional distress, reduced motivation, and cognitive impairments. The elaboration of the emotional dimension of pain requires cognitive processing, engaging multiple cortical and limbic regions involved in perception, modulation, and processing. Moreover, critical cognitive functions such as memory and attention are often disrupted in individuals experiencing chronic pain. In addition, mood disorders (e.g., depression) and anxiety disorders, frequently co-occur in chronic pain populations, highlighting the psychological comorbidity of the condition.

Recent research has shed light on key neural circuits and signaling pathways that contribute to the affective and cognitive components of pain (Corder et al., 2019; Massaly et al., 2019; Talbot et al., 2019; Caputi et al., 2019; Markovic et al., 2021; Choi et al., 2025) leading to the identification of novel therapeutic targets.

This Research Topic aims to describe different signaling pathways implicated in chronic pain and to offer possible novel therapeutic approaches that might pave the way to the management of pain disorders.

By using a spinal nerve ligation (SNL) model, the original research paper proposed by Mazzitelli et al. investigates the role of astrocytes in the amygdala’s central nucleus (CeA). They observed increased astrocyte activation at the chronic (4 weeks post-SNL) but not acute (1 week post-SNL) phase. Inhibiting astrocytes with fluorocitric acid (FCA) enhanced neuronal excitability by altering hyperpolarization-activated current without affecting synaptic transmission at the parabrachial nucleus-CeLC synapse. Behavioral tests showed that FCA influenced mechanical withdrawal thresholds and evoked vocalizations but did not alter facial grimacing or anxiety-like behaviors. These findings indicate astrocytes in the CeA may have protective functions in chronic neuropathic pain.

Cai et al. explored the dorsal medial prefrontal cortex (dmPFC) to basolateral amygdala (BLA) pathway’s role in emotional regulation. Optogenetic activation of this circuit in normal rats induced anxiety- and depression-like behaviors, while inhibition reduced anxiety and promoted reward-seeking behaviors. Their findings indicate the dmPFC-BLA circuit plays a critical role in mood regulation, with implications for pain-related emotional dysregulation.

Several key brain areas and neurotransmitters mediate emotional and cognitive aspects of pain. Flores-Garcia et al. reviewed the ventral tegmental area (VTA) as a hub integrating pain and emotional processing. Mounting evidence suggests that the dopaminergic system within the VTA plays a pivotal role in chronic pain’s affective components. The review emphasizes the importance of animal models incorporating both pain and mood disorder features to develop effective treatments. A review by Lançon and Seguela examined the anterior cingulate cortex (ACC) in top-down modulation of pain perception, focusing on monoaminergic pathways—dopamine (DA), norepinephrine (NE), and serotonin (5-HT). Chronic pain disrupts these neuromodulators, contributing to hyperexcitability and cognitive impairments. Understanding how these alterations affect pain perception could pave the way for novel, non-opioid-based therapies.

Marino et al. used a machine learning (ML) approach to analyze central μ-opioid (μOR) and dopamine D2/D3 (DOR) receptor profiles in chronic migraine, a debilitating neurovascular pain disorder with a significant impact on cognitive and emotional functions. Using Compressive Big Data Analytics on data from positron emission tomography scans, they distinguished migraine patients from healthy controls with over 90% accuracy by identifying key predictive regions from each receptor system. For μOR, these regions included the anterior insula, thalamic nuclei, and putamen, while DOR dysfunction was primarily identified in the putamen. These findings offer insights into the neurochemical disruptions underlying migraine-related pain and cognitive impairments in humans.

Corasaniti et al. assessed the efficacy of monoclonal antibodies (mAbs) targeting the calcitonin gene-related peptide (CGRP) or its receptor (CGRP-R) in treatment-resistant chronic migraine. Their real-world data suggest that combining anti-CGRP therapy with onabotulinumtoxin A reduces monthly headache days by ≥ 50% in 58.8% of patients. Erenumab, when combined with onabotulinumtoxin A, improved symptoms in 65% of cases, while eptinezumab was notable for its rapid onset. These findings emphasize the potential of combination therapy for improved migraine management.

A review by Alorfi reviewed drugs tested in completed phase IV clinical trials for fibromyalgia. Analyzing 1,263 trials (121 related to fibromyalgia), they identified 10 meeting inclusion criteria. Investigated drugs targeting primary pain and cognitive-emotional pathways included milnacipran, duloxetine, pregabalin, tramadol-acetaminophen, and armodafinil. Despite these trials, current pharmacological treatments remain of limited effectiveness, emphasizing the need for alternative therapeutic strategies.

Liu et al. conducted a Mendelian randomization analysis to explore the causal link between mood instability and chronic low back pain. Their findings demonstrated significant associations, with inverse variance weighting revealing odds ratios above 3.0, reinforcing the role of emotional dysregulation in chronic pain susceptibility.

The issue is concluded with a study by Lopez-Cordoba et al. that investigated spinal α2-adrenoceptor subtypes in nociception. Their results indicated that α2A-adrenoceptor activation contributes to antinociception in acute and tonic pain, while α2C-adrenoceptors may be pronociceptive under tonic nociceptive conditions, possibly by inhibiting GABAergic transmission. These findings highlight spinal adrenergic systems as potential therapeutic targets.

Collectively, these studies underscore the multifaceted nature of chronic pain, integrating neuropathic pain, migraine, fibromyalgia, and low back pain with cognitive and emotional processing. Key brain regions—including the amygdala, VTA, mPFC, and ACC—alongside neurotransmitter-receptor systems such as CGRP-R, D2/D3, μOR, and α2-adrenoceptors, play paramount roles in pain perception, mood regulation, and cognitive function. Advances in understanding these mechanisms may facilitate targeted therapies addressing both sensory and affective pain components. Furthermore, pharmacological innovations, including monoclonal antibodies, botulinum toxin, and novel neuromodulators, hold promise for treatment-resistant conditions. A multidisciplinary approach targeting brain, spinal, and neurochemical systems, could offer more effective and comprehensive strategies for managing chronic pain and its associated cognitive and emotional dysfunctions.
